# Prevalence and Causes of Prescribing Errors: The PRescribing Outcomes for Trainee Doctors Engaged in Clinical Training (PROTECT) Study

**DOI:** 10.1371/journal.pone.0079802

**Published:** 2014-01-03

**Authors:** Cristín Ryan, Sarah Ross, Peter Davey, Eilidh M. Duncan, Jill J. Francis, Shona Fielding, Marie Johnston, Jean Ker, Amanda Jane Lee, Mary Joan MacLeod, Simon Maxwell, Gerard A. McKay, James S. McLay, David J. Webb, Christine Bond

**Affiliations:** 1 School of Pharmacy, Queen's University Belfast, Belfast, United Kindgom; 2 School of Medicine and Dentistry, University of Aberdeen, Aberdeen, United Kingdom; 3 School of Medicine, University of Dundee, Dundee, United Kingdom; 4 Health Services Research Unit, University of Aberdeen, Aberdeen, United Kingdom; 5 Health Services Research and Management Division, City University London, London, United Kingdom; 6 Medical Statistics Team, University of Aberdeen, Aberdeen, United Kingdom; 7 Health Psychology, University of Aberdeen, Aberdeen, United Kingdom; 8 Clinical Pharmacology Unit, University of Edinburgh, Edinburgh, United Kindgom; 9 Department of Clinical Pharmacology, Glasgow Royal Infirmary, Glasgow, United Kindgom; 10 Clinical Pharmacology Unit, University of Edinburgh, Royal Infirmary of Edinburgh, Edinburgh, United Kindgom; 11 Centre of Academic Primary Care, University of Aberdeen, Aberdeen, United Kingdom; Charité University Medicine Berlin, Germany

## Abstract

**Objectives:**

Study objectives were to investigate the prevalence and causes of prescribing errors amongst foundation doctors (i.e. junior doctors in their first (F1) or second (F2) year of post-graduate training), describe their knowledge and experience of prescribing errors, and explore their self-efficacy (i.e. confidence) in prescribing.

**Method:**

A three-part mixed-methods design was used, comprising: prospective observational study; semi-structured interviews and cross-sectional survey. All doctors prescribing in eight purposively selected hospitals in Scotland participated. All foundation doctors throughout Scotland participated in the survey. The number of prescribing errors per patient, doctor, ward and hospital, perceived causes of errors and a measure of doctors' self-efficacy were established.

**Results:**

4710 patient charts and 44,726 prescribed medicines were reviewed. There were 3364 errors, affecting 1700 (36.1%) charts (overall error rate: 7.5%; F1:7.4%; F2:8.6%; consultants:6.3%). Higher error rates were associated with : teaching hospitals (p<0.001), surgical (p = <0.001) or mixed wards (0.008) rather thanmedical ward, higher patient turnover wards (p<0.001), a greater number of prescribed medicines (p<0.001) and the months December and June (p<0.001). One hundred errors were discussed in 40 interviews. Error causation was multi-factorial; work environment and team factors were particularly noted. Of 548 completed questionnaires (national response rate of 35.4%), 508 (92.7% of respondents) reported errors, most of which (328 (64.6%) did not reach the patient. Pressure from other staff, workload and interruptions were cited as the main causes of errors. Foundation year 2 doctors reported greater confidence than year 1 doctors in deciding the most appropriate medication regimen.

**Conclusions:**

Prescribing errors are frequent and of complex causation. Foundation doctors made more errors than other doctors, but undertook the majority of prescribing, making them a key target for intervention. Contributing causes included work environment, team, task, individual and patient factors. Further work is needed to develop and assess interventions that address these.

## Introduction

Prescribing errors are known to account for a substantial proportion of all medication errors and are an important cause of harm to patients [1], making them a priority area for patient safety initiatives. As the majority of prescribing in secondary care is undertaken by junior doctors, this group has been highlighted as a target group for educational interventions.

Two recent systematic reviews have reported on the prevalence of prescribing errors. However, both noted that a lack of consistency in study design, data collection methods and definitions of errors contributed to a wide variation in the error rates reported [2], [3]. Lewis *et al.* reviewed 65 studies of errors made by all groups of doctors and reported a median prescribing error rate of 7% (IQR 2–14) of items prescribed, 52 (IQR 8–227) errors per 100 admissions and 24 (IQR 6–212) errors per 1000 patient days [2]. Ross *et al.* reviewed 24 studies focussing on those doctors below consultant grade and reported an error rate of 2–514 per 1000 items prescribed and 4–82% of patients or prescription charts reviewed [3].

More recently, the EQUIP study, conducted in 20 English hospitals (at the same time as the study reported in our paper), reported a prescription error rate of 8.9% for all medication orders [4]. The error rate amongst junior doctors in their first two years of postgraduate training (F1 and F2) was significantly greater (8.4% and 10.3% for F1 and F2 respectively), than that of their senior colleagues (5.9% for hospital consultants).

Although the majority of prescriptions are written by junior doctors, few studies have focused primarily on junior doctors and their prescribing errors. We undertook the PROTECT (PRescribing Outcomes for Trainee Doctors Engaged in Clinical Training) study, to inform the development and delivery of intervention studies aimed at improving prescribing by junior doctors in Scotland. The aim was to determine the prevalence and perceived causes of prescribing errors made by junior doctors, and describe their knowledge, experience of prescribing errors and self-efficacy (i.e. confidence) in prescribing. Self-efficacy is defined as people's beliefs about their capabilities to produce designated levels of performance [5]. For the purposes of this study, junior doctors were defined as doctors in either their first foundation (F1) or second foundation (F2) year of post-graduate training).

## Methods

### Design

A three-part mixed-methods design was used, comprising: an observational study of the prevalence of prescribing errors (Study 1); semi-structured interviews with foundation doctors who had made prescribing errors (Study 2); a cross-sectional survey of foundation doctors (Study 3).

### Study 1 and Study 2

#### Participants and Setting

Studies 1 and Study 2 were conducted in a purposively selected sample of eight hospitals in Scotland. The participants were all grades of doctors prescribing in the study hospitals

#### Recruitment of hospitals and wards

Hospitals employing at least 12 F1s were approached sequentially by email to Health Board Directors of Pharmacy, and Chief Hospital Pharmacist. Eight hospitals were recruited, comprising one teaching hospital (TH; hospitals directly affiliated with a medical school) and one district general hospital (DGH; hospitals not directly affiliated with a medical school) from each of the four postgraduate areas in Scotland. Consent to recruit hospital medical and pharmacy staff to the study was obtained from both the Medical Director and Chief Pharmacist for each hospital site.

The main researcher (CR) visited all hospitals to explain the study to pharmacy staff. All hospital doctors were informed of the study by their Medical Director. Foundation doctors joining the hospitals during the study period were informed of the study by their educational supervisors.

The study was undertaken in purposively selected wards in each hospital, to ensure inclusion of a range of adult medical, surgical, acute and long stay patients. For inclusion, wards had to have at least one prescribing F1 doctor and a routine clinical pharmacy service. Paediatric and obstetric units were excluded, as often F1 doctors do not prescribe in these specialities.

#### Definitions

We adopted Dean's definition of a prescribing error: “one which occurs when, as a result of a prescribing decision or prescription writing process, there is an unintentional significant reduction in the probability of treatment being timely and effective or an increase in the risk of harm when compared with generally accepted practice” [6]. This definition excludes a number of behaviours such as prescribing a non-stocked medication and non-generic prescribing.

#### Data collection: Study 1: Prospective observational study

Following a comprehensive pilot, data collection started in March 2010, and continued for 14 months, which permitted exploration of longitudinal trends during one complete training year and comparison across two foundation year cohorts. In each study hospital, data were collected from each participating ward/unit for one week of each calendar month equating to a total of 28 ward weeks per hospital.

As per usual local practice, ward clinical pharmacists reviewed prescription charts for possible errors and for study purposes, recorded data on: age, sex, allergy status, number of medicines prescribed, grade of prescribing doctor. For identified errors, the date, time, stage of patient stay and error details were recorded. Forms were returned to the researchers who categorised errors by type, based on a classification system derived from a combination of the literature and our previous work. (1,6)

Reliability of error reporting was checked in a 10% random sample of cases, with and without errors, by the main researcher (CR). Potential harm resulting from the errors was classified by the research team using the NCCMERP (National Coordinating Council for Medication Error Reporting) system [7].

#### Data collection: Study 2: Interview Study

During each observation week in Study 1, all identifiable foundation doctors who had made an error were contacted by the ward pharmacist within 96 hours of prescription writing, given an information leaflet and invited to participate in a semi-structured interview about the error with the main researcher (CR). They were assured that all information would be treated in complete confidence. Contact details of those agreeing to participate and details of the error(s) were sent to the researcher. Interviews conducted either face to face, or by telephone, were recorded, transcribed, and analysed using content analysis. The types of errors and the perceived causes of errors were described using Reason's Model of Accident Causation and human error and errors described classified according to type (slip, lapse, mistake and violation) in line with the theory [8]. Full details of the process are reported elsewhere [9].

### Study 3: Cross Sectional Study

#### Participants and Setting

All F1 (n = 781) and F2 (n = 783) doctors working in Scotland in 2010 were eligible to participate.

#### Questionnaire development and administration

The questionnaire included questions on: doctor demography, space for description of an error made by the respondent (free text), scaled responses to a series of statements classifying the causes of that error (based on Reason's Model of Accident Causation and Human error [8], and questions on self-efficacy in conducting various prescribing tasks e.g. deciding on the most appropriate dose, based on Bandura's Social Cognitive Theory [10]. Full details are reported elsewhere [11].

The questionnaire was piloted both as a paper version and as a weblink. The weblink was sent by NHS Education Scotland (NES) to middle grade doctors on our behalf. Both versions of the questionnaire were refined post pilot. The questionnaire was distributed at the beginning of training seminars organised for foundation doctors in each of the hospitals participating in Study 1. Questionnaires were also available to complete on line. All questionnaires had an initial screening question to minimise duplication across distribution methods.

#### Statistical power and analyses

We based our statistical power calculation for Study 1 on the following conservative estimates: wards have an average of 20 beds, the average patient stay is one week and each patient is prescribed an average of five medications. We estimated there would be 4,480 patients and 22,400 items prescribed in participating wards during the 14-month study period. With 22,400 items, the 95% confidence interval for a prevalence of prescribing errors of 15% is 14.5% to 15.5%.

The following analyses were conducted for Study 1: the overall prevalence of prescribing errors by doctors per medication item written, and per patient, by hospital type and by grade of doctor. The associations between the prevalence and number of errors with postgraduate training year were assessed using the Mann-Whitney test and Chi squared ((χ^2^) test. Poisson regression was used to identify independent predictors of error frequency with rate ratios (95% confidence intervals) being calculated. The models were then adjusted for year cohort, patient gender, and month of data collection, a measure of patient turnover, hospital type and ward type. For Study 2, the semi-structured interviews were analysed using content analysis and Reason's Model of Accident Causation and Human Error [8]. For study 3, the Chi-squared (χ^2^) test was used to assess the association between the perceived causes of prescribing errors and year of training (F1 or F2).

#### Ethical approval

Approval for all aspects of the programme was granted by the North of Scotland Research Ethics Committee.

## Results

### Response rates

Ten hospitals were approached, and nine hospitals took part in the prevalence and interview studies; in one post graduate training area, two THs divided the data collection equally between them, to minimise the additional work for their pharmacists. Two hundred and one (90%) of the planned data collection weeks were completed. One hospital withdrew after six months of data collection. Of the remaining sites, five completed all 28 data collection weeks. A total of 4710 patients, and 44726 prescribed items were reviewed.

Pharmacists provided contact details for 54 doctors who had made an error; 40 doctors (31 F1s and 9 F2s) making one hundred errors were contacted and interviewed (14 face-to-face and 26 telephone). The remaining 14 doctors were either un-contactable, or when contacted, were unable to participate in the study, due to work or annual leave commitments. One interview accounted for 16 different errors (medicines prescribed for the wrong patient). Fourteen doctors were not interviewed due to their working schedules.

548 completed questionnaires were returned equating to 35.0% (548/1564) of the national cohort, and around 90% of those approached directly. The majority of respondents were F1s (64.4% (353)), female (58.9% (323)) and Scottish graduates (79.9% (438)).

In the following sections findings from the individual studies are integrated under the study objectives. Full reports of all the findings from Study 2 and 3 are reported separately [9], [11].

### Prevalence of prescribing errors

Prescribing errors were found in 36% (1700/4710) of patient prescription charts and 7.5% (3364/44726) of items prescribed. The error rate per patient was significantly greater in THs (1083/2622 (41.3%)), than in DGHs (617/2088 (29.5%)) p<0.001). A similar pattern was observed for error rates by item (Table 1).

**Table 1:** The overall error rate by prescribed item, per prescriber's grade overall and per hospital type (based on the 4820 reviews).

	Overall	F1	F2	Staff Grade	SHO/ST/SpR	Consultant	Non medical prescribers	Unknown	p-value
**Overall**									
Total number of prescriptions written	44726	23294	5329	613	7203	1423	360	6504	
Percentage of total		52.1	11.9	1.4	16.1	3.2	0.80	14.5	
Total number of errors	3364	1725	461	25	636	89	19	409	
Percentage of total		51.3	13.7	0.7	18.9	2.6	0.6	12.2	
Error rate (%)	7.5%	7.4%	8.6%	4.1%	8.8%	6.3%	5.3%	6.3%	<0.001
**Teaching Hospitals**									
Total number of prescriptions written	24898	12580	3795	131	3321	488	168	4415	
Percentage of total		50.5	15.2	0.5	13.3	2.0	0.7	17.7	
Total number of errors	2310	1187	354	11	371	41	15	331	
Percentage of total		51.4	15.3	0.5	16.1	1.8	0.6	14.3	
Error rate (%)	9.3%	9.4%	9.3%	8.4%	11.1%	8.4%	8.9%	7.5%	<0.001
**District General Hospitals**									
Total number of prescriptions written	19828	10714	1534	482	3882	935	192	2089	
Percentage of total		54.0	7.7	2.4	19.6	4.7	1.0	10.5	
Total number of errors	1054	538	107	14	265	48	4	78	
Percentage of total		51.0	10.2	1.3	25.1	4.6	0.4	7.4	
**Error rate (%)**	5.3%	5.0%	7.0%	2.9%	6.8%	5.1%	2.1%	3.7%	<0.001

F1: Doctors in their first year of post-graduate training; F2: Doctors in their second year of post-graduate training; SHO: Senior House Officer; ST: Speciality Trainee; SpR: Specialist registrar.

The most commonly encountered error type was medication omitted, 28.6% (963/3364) and this occurred significantly more frequently in THs (p<0.001)) (Table 2). The omission of prescriber's signature and the use of an inappropriate abbreviation were encountered more frequently in DGHs (p<0.001). When considering the self-reported errors described by questionnaire respondents, the most frequently mentioned error type was omission of medication (24%).

**Table 2:** The type of errors encountered overall and per type of hospital.

Type of error	Overall (n = 3364) n (%)	TH (n = 2310) n (%)	DGH (n = 1054) n (%)	p-value*	Interview errors (n = 100;%)
Medication omitted	963 (28.6)	719 (31.1)	244 (23.1)	<0.001	24 (24)
Incomplete prescription	527 (15.7)	338 (14.6)	189 (17.9)	0.017	6 (6)
**Incorrect dose:**					15 (15)
sub therapeutic	261 (7.8)	168 (7.3)	93 (8.8)	0.136	
supra-therapeutic	173 (5.1)	136 (5.9)	37 (3.5)	0.005	
**Incorrect frequency:**					12 (12)
correct total daily dose	21 (0.6)	16 (0.7)	5 (0.5)	0.610	
incorrect total daily dose	238 (7.1)	160 (6.9)	78 (7.4)	0.671	
Medication prescribed without indication	181 (5.4)	132 (5.7)	49 (4.6)	0.235	3 (3)
Duplication of therapy	154 (4.6)	87 (3.8)	67 (6.4)	0.001	1 (1)
Inappropriate abbreviation	148 (4.4)	72 (3.1)	76 (7.2)	<0.001	-
Incorrect timing	117 (3.5)	78 (3.4)	39 (3.7)	0.709	9 (9)
Omission of prescribers signature	88 (2.6)	42 (1.8)	46 (4.4)	<0.001	-
Incorrect formulation	84 (2.5)	70 (3.0)	14 (1.3)	0.005	3 (3)
Illegible	66 (2.0)	52 (2.3)	14 (1.3)	0.098	-
Missing Instructions for use	32 (2.8)	4 (0.4)	28 (1.2)	0.034	-
Incorrect drug	57 (1.0)	39 (1.7)	18 (1.7)	1.000	4 (4.0)
Significant drug-drug interaction	51 (1.5)	32 (1.4)	19 (1.8)	0.443	2 (2.0)
Incorrect route	40 (1.2)	27 (1.2)	13 (1.2)	1.000	1 (1.0)
Incorrect duration	37 (1.1)	26 (1.1)	11 (1.0)	0.974	1 (1.0)
Contra-indication to medication	32 (1.0)	27 (1.2)	5 (0.5)	0.083	3 (3.0)
**Wrong patient**	16 (0.5)^1^	16 (0.7)	0 (0)	0.015	16 (16.0)
**Patient allergic to medication prescribed**	16 (0.5)	10 (0.4)	6 (0.6)	0.793	-
**Other**	62 (1.8)	35 (1.5)	27 (2.6)	0.051	

TH: Teaching Hospitals, DGH: District General Hospitals;

^1^ occurred for one single patient who had attached to their name a list of prescriptions for someone else.

comparing between hospitals the error rate for each reason out of total number of errors.

With respect to error theory classification, slips (n = 222; 43.7%) and mistakes (n = 111; 21.9%) accounted for the majority of self -reported errors in the questionnaires. The same pattern was observed when the interview data was analysed with slips (n = 30; 30%) and mistakes (n = 18; 18%) being more common than lapses (n = 11; 11%) and violations (n = 6; 6%). For both the questionnaire and the interview studies, there were several instances where the error types, reported or observed, could not be classified according to HET (n = 111; 21.9% and n = 35; 35% respectively). This was largely due to a lack of information provided by the respondent, or the fact that the error observed was one which had originated from a different prescriber.

The majority of errors occurred at time of admission to hospital (1907; 56.7%) (Table 3). Significantly higher error rates were associated with being: in the first cohort of data collection (up to July 2010); on a surgical or mixed ward compared to a medical ward; in a TH compared to a DGH in a ward with a higher turnover of patients, and having a higher total of number of medications prescribed (Table 4).

**Table 3:** Stage of Hospital Stay when errors occur.

Stage of Hospital Stay	Total* (N = 3364) n (%)	TH (N = 2310) n (%)	DGH (N = 1054) n (%)	p-value**
Admission	1907 (56.7)	1403 (60.7)	504 (47.8)	<0.001
Transcription of a new drug chart	123 (3.7)	74 (3.2)	49 (4.6)	0.049
Discharge	489 (14.5)	308 (13.3)	181 (17.2)	0.004
Remainder of inpatient Stay	825 (24.5)	514 (22.3)	311 (29.5)	<0.001
While Decanting	12 (0.4)	3 (0.1)	9 (0.9)	0.002 (FE)
Other/unknown/not specified	38 (1.1)	25 (1.1)	13 (1.1)	0.835

TH: Teaching Hospital; DGH: District General Hospital.

More than one option could be selected.

pairwise chi-squared.

**Table 4:** Poisson regression for number of errors.

		Unadjusted Analysis			Adjusted Analysis^1^			Adjusted Analysis^2^		
		Rate Ratio	95% CI	p-value	Rate Ratio	95% CI	p-value	Rate Ratio	95% CI	p-value
**Cohort**	Up to July 2010	1.00			1.00			1.00		
	August 2010 onwards	0.86	(0.81, 0.93)	<0.001	0.87	(0.79, 0.97)	0.01	0.9	(0.81, 1.00)	*0.056*
**Gender**	Male	1.00			1.00			1.00		
	Female	1.07	(1.00, 1.15)	0.047	1.08	(1.01, 1.15)	*0.033*	1.043	(0.97, 1.12)	0.226
**Ward Type**	Medical	1.00			1.00			1.00		
	Surgical	1.02	(0.95, 1.09)	0.656	1.11	(1.03, 1.19)	*0.009*	1.14	(1.06, 1.23)	<0.001
	Both	1.03	(0.75, 1.41)	0.855	1.41	(1.01, 1.95)	*0.041*	1.57	(1.13, 2.18)	0.008
**Hospital Type**	DGH	1.00			1.00			1.00		
	TH	1.77	(1.65, 1.91)	*<0.001*	1.83	(1.69, 1.98)	*<0.001*	1.82	(1.68, 1.97)	*<0.001*
**Total Medicines**	Per additional Med	1.05	(1.04, 10.6)	*<0.001*				1.05	(1.04, 1.06)	*<0.001*
**Patient Turnover**	<26	1.00			1.00			1.00		
(Average per 5 days)	> = 26 to <35	1.18	(1.05, 1.31)	*0.004*	1.05	(0.94, 1.18)	0.366	1.04	(0.92, 1.15)	*0.593*
	> = 35 to <48	1.42	(1.27, 1.58)	*<0.001*	1.09	(0.97, 1.21)	0.156	1.08	(0.97, 1.21)	*0.163*
	>48	1.50	(1.35, 1.66)	*<0.001*	1.18	(1.06, 1.32)	*0.002*	1.21	(1.09, 1.35)	*<0.001*
**Month of**	August	1.00			1.00			1.00		
**Data Collection**	September	0.70	(0.56, 0.89)	*0.003*	0.74	(0.58, 0.94)	*0.013*	0.77	(0.61, 0.98)	*0.031*
	October	0.99	(0.82, 1.18)	0.885	0.85	(0.70, 1.01)	0.071	0.84	(0.70, 1.01)	0.057
	November	1.08	(0.89, 1.31)	0.448	0.98	(0.81, 1.20)	0.874	0.96	(0.79, 1.17)	0.718
	December	1.66	(1.38, 2.00)	*<0.001*	1.56	(1.29, 1.88)	*<0.001*	1.63	(1.35, 1.97)	*<0.001*
	January	1.06	(0.86, 1.30)	0.587	0.84	(0.69, 1.04)	0.111	0.86	(0.70, 1.06)	0.17
	February	1.14	(0.95, 1.35)	0.15	0.97	(0.81, 1.16)	0.724	1.00	(0.84, 1.20)	0.975
	March	1.24	(1.05, 1.46)	*0.01*	1.03	(0.85, 1.23)	0.791	1.04	(0.87, 1.25)	*0.679*
	April	1.23	(1.06, 1.43)	*0.007*	1.04	(0.88, 1.23)	0.646	1.03	(0.86, 1.21)	*0.769*
	May	0.98	(0.82, 1.16)	0.78	0.89	(0.74, 1.08)	0.239	0.93	(0.76, 1.12)	0.437
	June	1.42	(1.22, 1.67)	*<0.001*	1.23	(1.03, 1.46)	*0.022*	1.33	(1.12, 1.58)	*0.001*
	July	1.41	(1.19, 1.67)	*<0.001*	1.13	(0.93, 1.37)	0.227	1.15	(0.95, 1.40)	*0.162*

^1^ not including total medicines;

^2^ including total medicine.

### Comparison of error rate by doctor grade

F1s were responsible for half (51.3%) of all errors, but were also responsible for half (52.1%) of all prescribing. The resultant error rate for F1s was 7.4% per item prescribed, for F2s 8.6%, for staff grades 4.1%, for speciality trainees 8.8%, and for consultants 6.3% (Table 1). In the questionnaire, 514 (93.8%) foundation doctors estimated their daytime error rate; F1 doctors estimated a significantly higher error rate (median 6.7; IQR 2–12.4) than F2 doctors (median 4; IQR (0–10) (p = 0.002).

### Perceived causes of error

**Figure 1:**
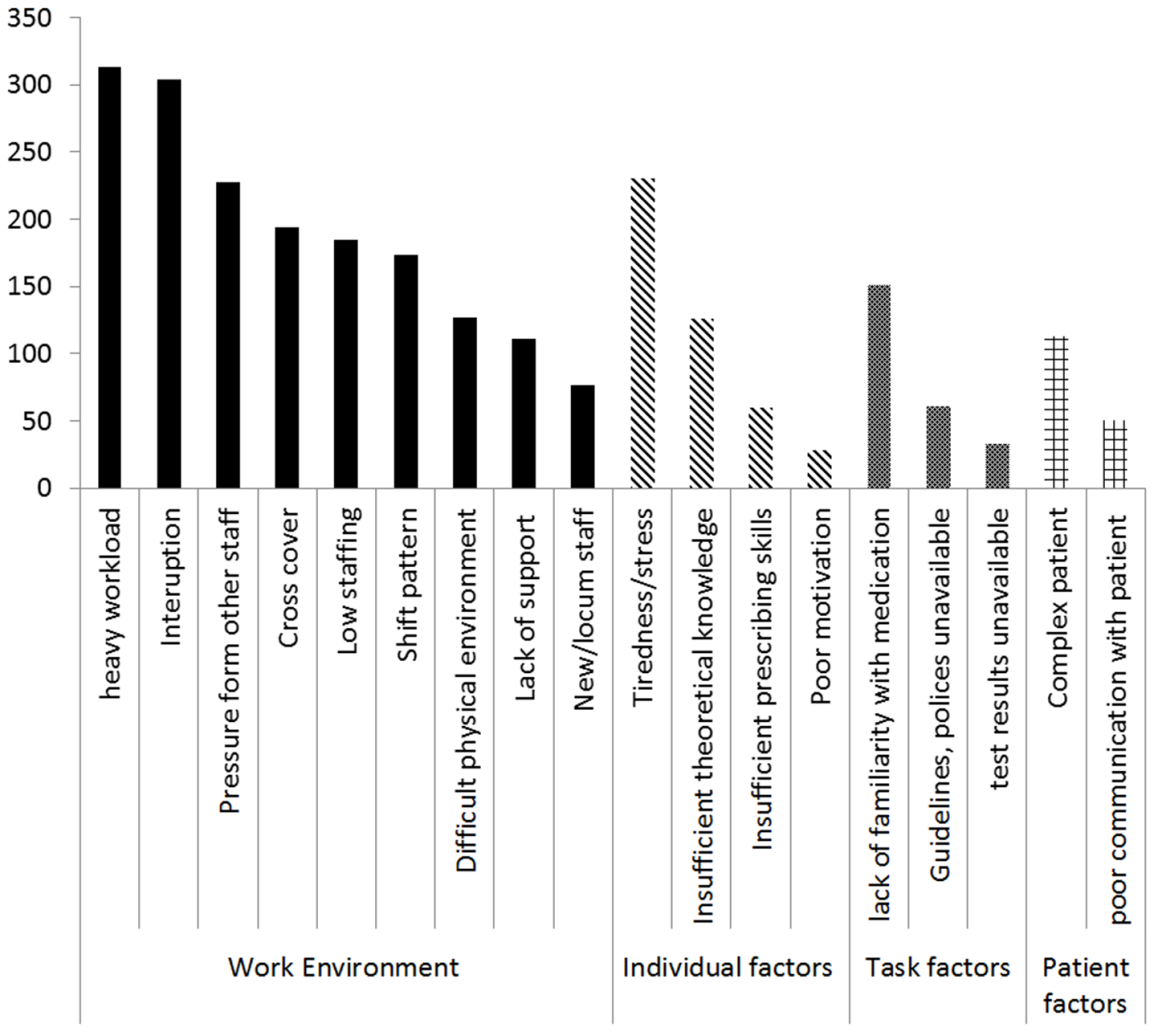
Frequency of reporting of suggested causes of prescribing error as per specified list (N = 504 errors). (Note more than option could be selected).

In both the interviews and the questionnaire, doctors identified multiple contributory factors for each error. The most frequently mentioned error causing factor was the working environment (See Figure 1). This was exemplified by interviewees commonly citing workload and time pressures, and questionnaire respondents most commonly citing pressure from other staff, workload and being interrupted as the causes of errors. The main *task* factor identified by interviewees was poor availability of drug information at admission (often out of hours) but this was not as strongly reflected in the questionnaire responses in which the main task factor reported was lack of familiarity with the medicine. *Team* factors were also mentioned, including poor quality of drug information, and the number of different individuals (and teams) involved with a patient's care pathway; this was also reflected in the questionnaire responses where over a quarter of respondents cited inadequate communication as a causative factor. In both the interview and the questionnaire components of the study there was a strong assumption that other team members would intercept any prescribing errors. The majority of interviewees cited the pharmacist as their main defence for identifying errors and preventing them reaching the patient (Box 1). None of the doctors interviewed had reported their error through the hospital reporting system and the questionnaire responses confirmed that medical staff was unlikely to complete an error reporting form. *Individual* factors identified in the interviews were lack of knowledge/experience (126/504; 25%). Questionnaire respondents particularly highlighted tiredness and stress (230/504; 45.6%). The most frequently stated *patient* factor was complexity (e.g. polypharmacy) (113/504; 22.4%). Interviewees also indicated that they considered “*prescribing…. a low priority task – juniors should not change prescriptions made by other staff*”.

### Consequences of errors

**Figure 2:**
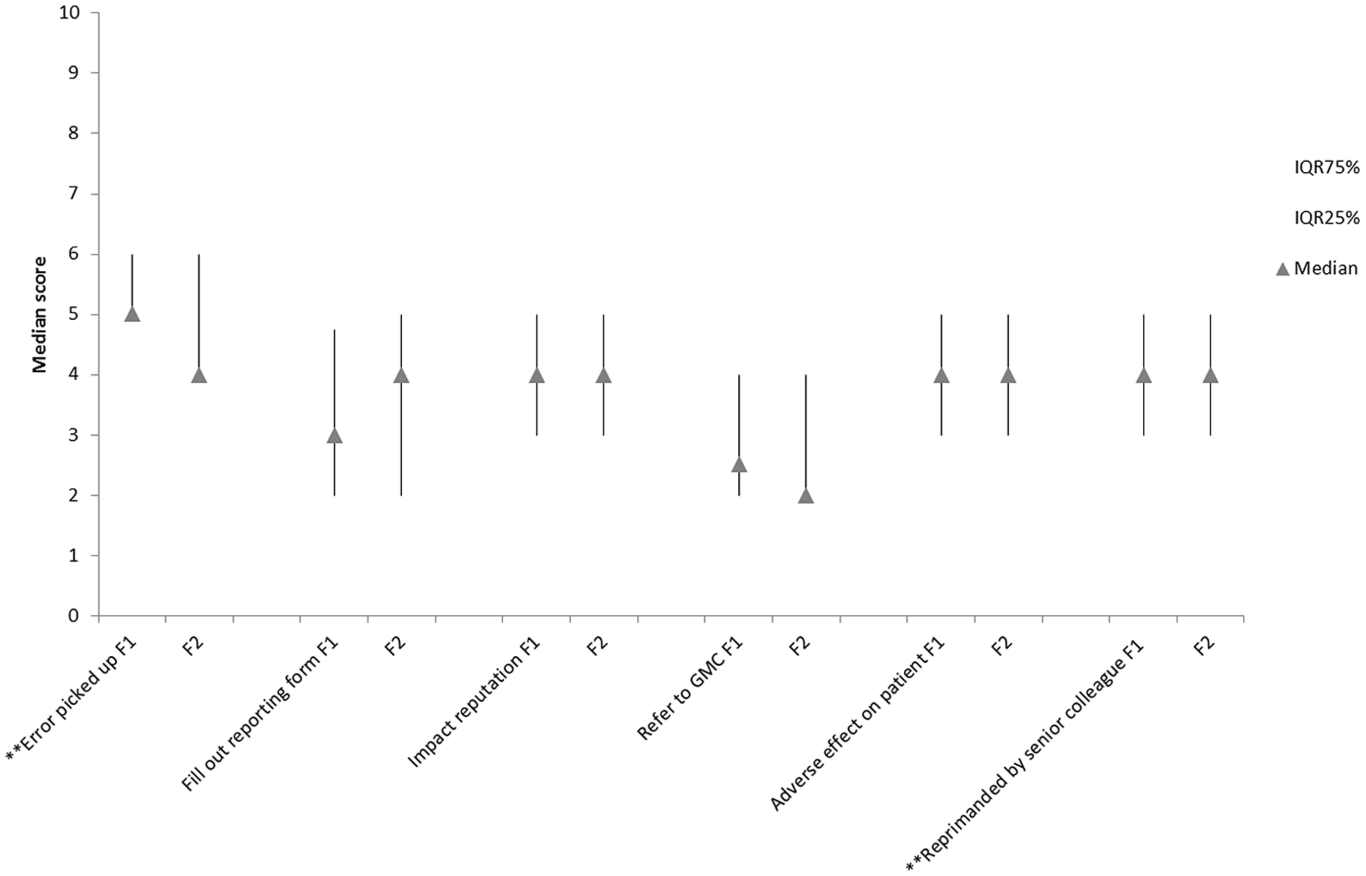
Median responses of questionnaire F1 (n = 353) and F2 (n = 323) respondents to series of statements on consequences of prescribing (note vertical lines represent the IQR. ** denotes statistical difference between F1 and F2 responders <0.05.

In the observational prevalence study, 60% of errors reached the patient, of which less than 1.0% caused actual harm or required monitoring. Of the errors reported by the questionnaire respondents, 32.4% reached the patient, and 12.6% of these (4.1% of all errors) may have caused some harm to the patient. Applying the NCCMERP taxonomy [7], 3.3% may have contributed to or resulted in temporary harm to the patient and required intervention, 0.6% may have resulted in prolonged hospitalisation and 0.2% in permanent patient harm. Referral to the General Medical Council and having to complete an error reporting form, were perceived as unlikely consequences of errors (Figure 2).

### Self-efficacy (confidence in prescribing) (Study 3)

**Figure 3:**
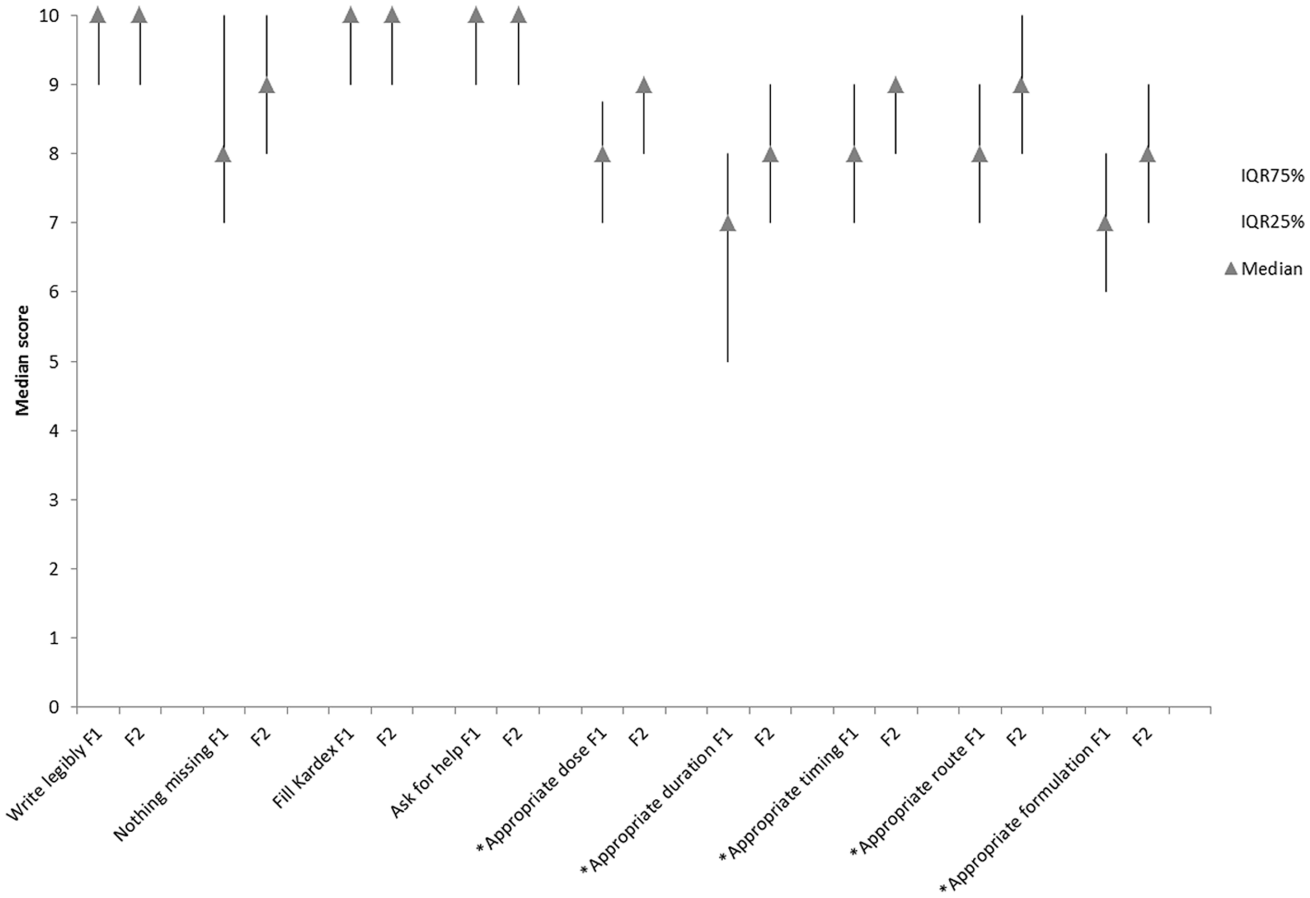
Median responses of questionnaire F1 (n = 353) and F2 (n = 323) respondents to series of statements on self-efficacy (confidence) in prescribing (note vertical lines represent the IQR. * denotes statistical difference between F1 and F2 responders <0.001.

Both F1 and F2 doctors reported being confident in the physical aspects of writing prescriptions (Figure 3). F1s had slightly less confidence in knowledge-based components, but nonetheless prescribing confidence was generally high. F2 doctors were significantly more confident than F1s in selecting the most appropriate dose, duration, timing and route.

## Discussion

### Main findings

Overall, 7.5% of prescribed items were associated with errors, affecting over a third of patients. Although error rates varied, error types were consistent across all doctor grades. Error rates for F1 doctors were significantly lower than F2s, but the highest error rate was observed for more experienced doctors in training, and lowest from those in staff grade posts. The prescribing error rate in District General Hospitals (DGHs) was significantly less than that in observed Teaching Hospitals (THs). Foundation doctors were generally both confident in their ability to prescribe and in their belief that if they made an error it would be picked up before it reached the patient. Challenges in the work environment were the most commonly cited reasons for error.

### Strengths and limitations

Our mixed-methods approach has several strengths. Both observational and self-report data are subject to a range of biases and the broadly similar results from different approaches give us greater confidence in the validity of our findings. We also minimised the biases inherent to each method; the use of ward pharmacists for data collection minimised the Hawthorne effect [12] as the impact of additional clinical surveillance by an independent researcher was avoided [13], and no new ward procedures were introduced. Doctors were interviewed as near to the time of their error as possible, thus minimising recall bias. The generalisabilty of the findings is strengthened by inclusion of a range of ward and hospital types from across Scotland and the use of a mixed approach to questionnaire distribution to maximise response rates. Furthermore the overall error rate reported in the current Scottish study is of the same order as that found in the recent EQUIP study undertaken in England [4]. The limitations of our study include variation in a clinical pharmacist's ability to identify errors, or to record all the errors identified, and the self-selected nature of those agreeing to be interviewed or return the questionnaire. Our initial power calculation was based on an estimated an error rate of 15% for 22,400 items. Whilst the actual error rate was lower than this (7.5%), the number of items was higher (44,726). A post-hoc sample size calculation gives a 95% confidence interval around the actual error rate prevalence of 7.3% to 7.7%..

### Interpretation

Prescribing errors most frequently occurred at the time of patient admission, reflecting the known difficulties experienced in establishing a patient's current medication. While the Scottish Patient Safety Programme has already targeted medicines reconciliation [14], the current data suggest that problems remain, although the slightly lower error rate for the later months of observation may reflect some improvements in this area. Interestingly, in the interview study, foundation doctors commented that the medicines reconciliation process was not well used by other hospital doctors. They also reported practical difficulties such as insufficient time to comply with the standard to use two reference sources to confirm a patient's current medications i.e. contacting other health-care professionals (e.g. general practitioners, community pharmacists). Although foundation doctors were aware of the existence of the emergency care summary (ECS), which contains this information, many stated that they were unable to access this information due to a hospital failure to supply appropriate passwords despite repeated requests. Although errors at the time of patient discharge occurred less frequently, doctors highlighted, in both interviews and questionnaire responses that they were under pressure to discharge patients quickly, despite having insufficient uninterrupted time to write the discharge prescriptions and lack of previous involvement in the patient's care.

Previous studies have emphasised the multifactorial nature of prescribing errors [1], [4], [15], [16]. A key finding from our results was that environmental factors, and in particular workload, interruptions, pressure from other staff, and a lack of time, are perceived by medical staff as major causes of error. This is supported by the higher error rate in teaching hospitals and wards with the highest turnover of patients. Differences between F1 and F2 doctors' responses to the questionnaire indicate that unlike initial lack of knowledge, these problems do not resolve with experience. Interaction between these environmental factors is likely to limit the reliability of observational comparison between different settings. For example, a recent UK study reported significantly higher error rates in medical *versus* surgical wards [17], which is the opposite of our finding (Table 4). This difference is probably explained by the fact that all of the medical wards in the previous study were acute medical admissions units. Nevertheless, like our study (Table 3), they reported that omission of medicines on admission was the commonest type of error [17]. The difference in the setting may explain why the error rate in that study (14.7%) was almost twice as high as in our study or in EQUIP [4], [17].

As our study shows, not all errors will result in patient harm; patient factors and checks within the system are all likely to affect final outcomes for the patient. However, given the volume of prescribing even a small percentage of errors that reach the patient is unacceptable in terms of population harm. As it is not possible to predict which errors will cause harm, the aim must be to minimise the prevalence of any error. To date, previous interventions to address prescribing errors have had mixed success [18]. This highlights the need for the adoption of a systematic approach to design an intervention, by following the Medical Research Council's framework for complex interventions [19]. This study is the first step of that process. On the basis of our findings an ideal intervention should address both the environmental and individual factors. One such intervention at ward level would be to change the ward environment to ensure that prescriptions could be written without interruption, especially on admission, as, in addition to enabling junior doctors to prescribe accurately, this might also persuade them that prescribing accurately was important and that errors were not acceptable or safe and enable them to resist interruption and pressure from other staff.

In this regard, the questionnaire responses demonstrated a high degree of misplaced confidence in prescribing skills amongst the respondents despite high error rates. Although junior doctors reported high levels of confidence about their ability to write safe prescriptions, our observational findings demonstrated that this confidence was frequently misplaced when operating within the current NHS environment, replicating the mismatch between confidence and competence found in other areas of healthcare [20], [21]. We believe that this is an issue that should be urgently addressed with better workplace feedback to individual doctors during the earlier years of postgraduate training and better aggregate reporting of errors to clinical groupings of junior and senior doctors. Much has been said about the poor knowledge and lack of preparedness for prescribing of new medical graduates [22], with final year medical students and first year graduates medical graduates reporting a lack of confidence in their ability to meet GMC competencies, due to a lack of learning and assessment relating to prescribing [23]. This was reflected in both the interview and questionnaire findings. A combined intervention with training in error causation and avoidance using behavioural change techniques [24], [25] rather than focussing solely on knowledge may be the optimum approach.

## Conclusions

Taken together with the EQUIP study, this work has confirmed a baseline prevalence of errors using a standard error definition. This will inform the overall design and scale of any subsequent intervention studies. We have also confirmed the multifactorial nature of error causation and quantified the major part that error-producing conditions, unrelated to the individual prescriber, have to play in this.

## Supporting Information

File S1 **Quotes from interviewees with regards to common defence mechanisms used.**
(DOCX)
