# Supplementary material for: Prevalence and Causes of Prescribing Errors: The PRescribing Outcomes for Trainee Doctors Engaged in Clinical Training (PROTECT) Study
Source: PLoS One. 2014 Jan 3;9(1):e79802. doi: 10.1371/journal.pone.0079802 (PMC3880263; doi:10.1371/journal.pone.0079802)
Supplement: File S1 — Quotes from interviewees with regards to common defence mechanisms used. (DOCX) [file pone.0079802.s001.docx]

**Box 1:** Quotes from interviewees with regards to common defence mechanisms used

| Uuh I think, I’m not too sure because, because (pharmacists name) checks everything I do so any mistake I make will be corrected before it reaches the patient. 030  Yeah. It happens that one FY1 is in charge of one ward so any patient discharged from that ward would be your job to prepare the discharge letter.  I guess the team that was involved includes pharmacists, and you know that any error should be picked up by the pharmacists in my mind, at the back of my mind, it’s a bit of a safety net thing, knowing that someone will check through my prescription before it reaches the patient.  029. |
| --- |
